# Films, Gels and Electrospun Fibers from Serum Albumin Globular Protein for Medical Device Coating, Biomolecule Delivery and Regenerative Engineering

**DOI:** 10.3390/pharmaceutics14112306

**Published:** 2022-10-27

**Authors:** Elahe Mahdipour, Kibret Mequanint

**Affiliations:** 1 Department of Chemical and Biochemical Engineering, The University of Western Ontario, London, ON N6A 5B9, Canada; 2 Department of Medical Biotechnology & Nanotechnology, Faculty of Medicine, Mashhad University of Medical Sciences, University Ave., Mashhad 9177948564, Iran

**Keywords:** globular albumin, gels, electrospinning, biomolecule and gene delivery, cell delivery

## Abstract

Albumin is a natural biomaterial that is abundantly available in blood and body fluids. It is clinically used as a plasma expander, thereby increasing the plasma thiol concentration due to its cysteine residues. Albumin is a regulator of intervascular oncotic pressure, serves as an anti-inflammatory modulator, and it has a buffering role due to its histidine imidazole residues. Because of its unique biological and physical properties, albumin has also emerged as a suitable biomaterial for coating implantable devices, for cell and drug delivery, and as a scaffold for tissue engineering and regenerative medicine. As a biomaterial, albumin can be used as surface-modifying film or processed either as cross-linked protein gels or as electrospun fibers. Herein we have discussed how albumin protein can be utilized in regenerative medicine as a hydrogel and as a fibrous mat for a diverse role in successfully delivering drugs, genes, and cells to targeted tissues and organs. The review of prior studies indicated that albumin is a tunable biomaterial from which different types of scaffolds with mechanical properties adjustable for various biomedical applications can be fabricated. Based on the progress made to date, we concluded that albumin-based device coatings, delivery of drugs, genes, and cells are promising strategies in regenerative and personalized medicine.

## 1. Introduction

**Figure 1:**
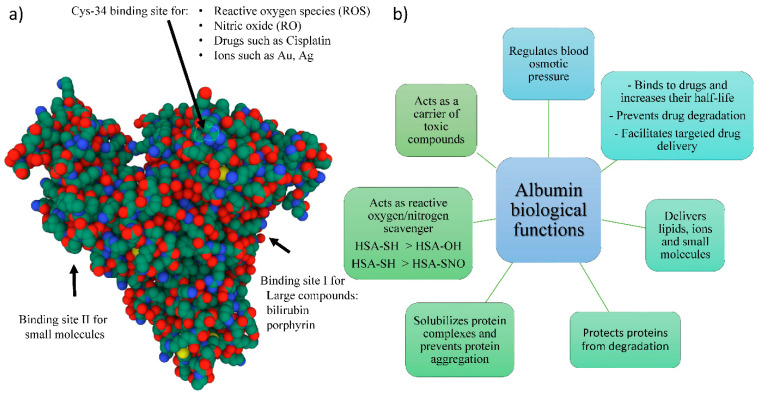
Albumin structure and functions. (**a**) Schematic presentation of different binding sites of albumin protein (3D structure is adapted from RCSB Protein Data Bank). Atoms are represented as spheres with conventional color coding: carbon (green), nitrogen (blue), oxygen (red), and sulfur (yellow). (**b**) Diverse biological functions of albumin. HAS-SNO: *S*-Nitrosylated Human Serum Albumin.

Abundantly found in vertebrate blood, albumin is a glycosylated and negatively charged globular single polypeptide chain protein having a 66.5 kDa molecular weight. Albumin proteins from different species, such as bovine serum albumin (BSA) and chicken egg albumin (ovalbumin) have similar structures and sequence homology to human serum albumin (HSA) (Figure 1a). Human serum albumin (HSA) is composed of three homologous domains (I-III), and each domain has two subdomains forming a heart-shaped protein. Subdomains IIA (the binding site I) and IIIA (the binding site II) form the main ligand-binding regions of HSA (Figure 1a) [1]. Albumin maintains the oncotic or colloid osmotic pressure of blood and acts as a carrier for various components such as fatty acids, calcium, zinc, copper, and toxic compounds such as bilirubin. It also binds to drugs such as Naproxen and Warfarin and controls drug half-life and activity in the body [2]. Furthermore, albumin has antioxidant properties and provides a source of thiols in serum that acts as reactive oxygen/nitrogen scavengers. Body fluids such as cerebrospinal fluids are also rich in albumin, and through the lymphatic system, albumin communicates between circulation and extracellular spaces such as skin and muscles [3]. Albumin has also been shown to act as a chaperon and prevent the aggregation of other proteins. Albumin binds to stressed proteins and makes stable and soluble complexes, thereby preventing their aggregation [4] (Figure 1b). Not surprisingly, albumin has attracted attention as a natural protein of choice in drug delivery and regenerative medicine studies. Albumin scaffolds are less sensitive to protease degradation than collagen and fibrin since specific protease known to degrade serum albumin or cross-linked albumin at a neutral pH of ~7.5 are generally lacking; therefore, they are more suitable scaffolds to deliver cells and active molecules [5]. Notwithstanding its increased use as a biomaterial, a broad review of emerging data on albumin is limited because of the narrowly defined scope of prior reviews (e.g., cell transplantation, therapeutic agent or drug carrier) [6,7,8]. Therefore, the objective of this article is to provide readers the broad utility of albumin for implant coating and regenerative therapeutics, and property relationships.

## 2. Albumin-Coated Implant Materials

**Figure 2:**
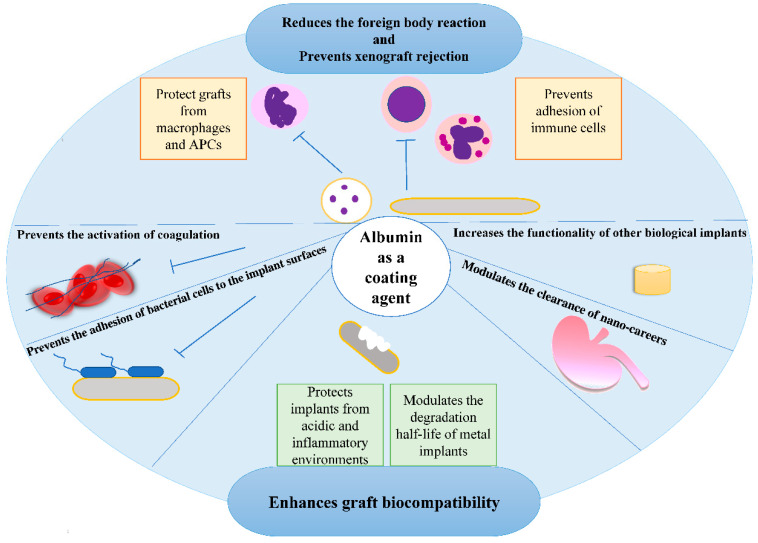
Advantages of albumin coatings on device biocompatibility and performance. When used as a coating agent, albumin can modulate the immune response and immune cell detection of xenografts, reduce coagulation, reduce bacterial and immune cell adhesion to implant surfaces, modulate the degradation and clearance of implants, and improve their functionalities.

One of the earliest applications of albumin was as a surface-modifying agent for blood-contacting synthetic biomaterials to improve their biocompatibility and bio-functionality (Figure 2). Cells and proteins do not attach to albumin in physiological conditions; thus, studies have used this property of albumin to reduce the adhesion of immune cells to implanted biomaterials [9]. Albumin coating of synthetic polyester vascular grafts enhanced their hemocompatibility by reducing the coagulation activation and the adhesion of leukocytes to the surface [10,11]. In addition, the albumin layer on implants reduces bacterial adhesion through charge repulsion between the negatively charged bacteria and the implant surface, thus minimizing device infection [12,13]. Coating the surface of nano-carriers with albumin could reduce the host’s response to nano-carriers, whereby denaturation of albumin structure at the surface of nano-carrier was introduced as a way to modulate the clearance rate of the nano-carrier as well as to control the detection of nano-carriers by macrophages [14]. Albumin was also used to cover the surfaces of xenografts for attenuation of acute immune and inflammatory responses [15]. Besides immune system evasion, the incorporation of albumin in implants can modulate their degradation half-life.

In addition to polymers, metallic implants have various applications in medicine, especially in orthopedics. Since the degradation of these materials is very slow, the addition of polymers or proteins on the surface of metal implants can enhance the degradation rate. Albumin, as a frequent protein in body fluids, has been shown to be able to increase the corrosion rate of iron biomaterials. Albumin has the affinity to bind iron, and adsorption of albumin onto the implant surface resulted in albumin-iron ion complexation as a mechanism to increase the corrosion and degradation of iron implants [16]. On the other hand, it was shown that coating the titanium implant surface with albumin increased the corrosion resistance of implants. Albumin could protect the implant surface from acidic and inflammatory environments [17]. In addition, albumin coated on the surface of titanium implants decreased the adherence of bacterial cells to the implant surfaces [18]. A combination of albumin with tannic acid was used as a coating layer to protect breast implants made of polycaprolactone from *Staphylococcus* infection [19]. The presence of albumin in biological implants may increase their functionality; for instance, adding albumin to fibrin hydrogel increased the hydrogel permeability and enhanced the osteogenic and angiogenic activities of osteoblast and endothelial cells, respectively [20]. Moreover, albumin is a reducing agent for metals such as gold (e.g., chloroauric acid, H[AuCl_4_]·3H_2_O), silver, and copper to prepare metal colloids and nanostructured materials. The mechanisms and applications of albumin as a metal-reducing agent have been reviewed in recent papers [21,22] and, the zwitterionic properties of albumin at the isoelectric point enable it to bind metal ions and act as a reducing agent [23,24,25]. This leads to metal nanoparticle formation where the albumin coats and stabilizes them [23,24,25]. Through coating and capping, albumin makes metal nanoparticles more biocompatible [23,24]. Additionally, the zwitterionic property of albumin facilitates the production of alloy nanoparticles such as AgAu and AgTiO2 [23,26].

## 3. Albumin-Derived Gels

### 3.1. Gelation Process and Mechanism

**Figure 3:**
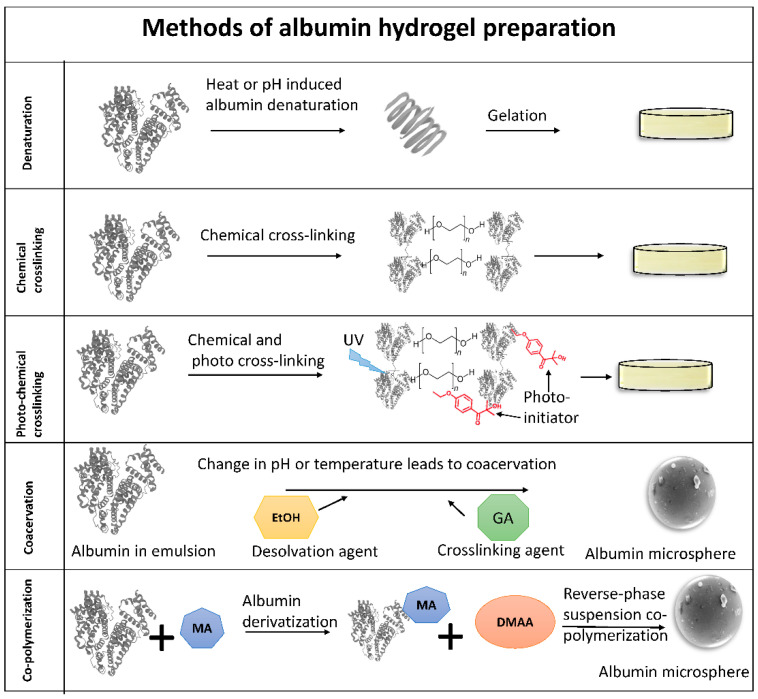
Albumin hydrogel fabrication and applications. Various strategies are available to induce gelation in albumin, and they have various applications in medicine. For chemical/photocrosslinking, albumin is first acylated and then the double bonds are cross-linked either thermally or photochemically. MA: methacrylic anhydride, DMAA: N,N-dimethyacrylamide, GA: glutaraldehyde, EtOH: ethanol, UV: ultraviolet.

Hydrogels are tunable three-dimensional cross-linked polymer networks that are hydrophilic and can absorb a large amount of water [27]. Protein hydrogels are smart materials as they can respond to external signals such as pH, temperature, and ionic strength [28]. Albumin hydrogels are transparent and porous, capable of absorbing water and encapsulating biomolecules. Studies have used various methods to create albumin-derived hydrogels, such as thermal denaturation, chemical cross-linking, irradiation, conjugates of polymer and albumin, nanostructures, and enzymatic methods (Figure 3). Heating induces albumin polymerization and self-crosslinking between albumin chains [29]; however, a temperature of (ca. 120 °C–140 °C) at alkaline pH reduced the tensile strength. In contrast, heating to 75 °C at pH 2 produced elastic albumin hydrogels [30]. Both heat and pH can initiate the polymerization of albumin, each with some limitations. A change in pH from 7.4, in which albumin has a natural heart shape structure, to pH 3.5 added positive charges on albumin and induced electrostatically partial denaturation of albumin, which in turn resulted in the self-assembly of albumin at a low temperature of 37 °C [31]. In fact, hydrophobic interactions at low pH (i.e., 3.5) resulted in albumin aggregation and hydrogel formation while intrinsic drug-binding properties of albumin were preserved, and such hydrogel can be used for controlled drug delivery [31]. Hydrogels formed through the electrostatically induced albumin self-assembly are more biodegradable compared with thermally denatured albumin gels [31]. At pH 7, incubation at a temperature above the denaturation temperature of albumin resulted in much faster (2 h) gelation compared with incubation at a temperature below the denaturation temperature (a week) [32]. Results from infrared spectroscopy revealed that the native secondary structure of albumin is well preserved when gelation occurs at low temperatures; however, these hydrogels formed at low temperatures were mechanically weak [32]. In fact, electrostatically induced gelation of albumin could occur at both low pH <4.3 and high pH >10.6 [32].

Reduction and changes in pH that resulted in the cleavage of disulfide bonds and the formation of free thiols could also be used as stimuli to induce albumin gel formation [33]. By removing the reducing agent, disulfide bridges can be reformed but in a non-native format and result in the denaturation of albumin and gel formation [33]. However, the gelation of albumin was affected by the albumin concentration and pH. An increase in albumin concentration (up to 750 µM) resulted in a uniform network structure of albumin hydrogel. A pH between 6 and 8 was reported to be optimum for the formation of intermolecular interactions, while no gel could be formed at alkaline pH > 9 [33].

Although successful gelation occurs at high temperatures, it also denatures the protein and may change the recognition sequences and binding sites for drugs or proteins. Moreover, heating reduces the transparency of albumin hydrogels. On the other hand, low or high pH may not be suitable for drug or cell encapsulation. Chemical cross-linking is another option to initiate gelation in albumin without heat and pH-related stimulation. Different end groups of polyethylene glycol (PEG) were used for albumin cross-linking, from which PEG-succinimidyl glutarate resulted in the successful formation of a degradable PEG-albumin scaffold [34]. Using a photo-initiator such as Irgacure TM2959 and UV light (365 nm) exposure, PEG-conjugate albumin was polymerized into hydrogels [35]. Crosslinking using ethylene glycol and UV irradiation was used to create cell adhesion and growth patterns on albumin-coated surfaces [36]. Glutaraldehyde is a common cross-linking agent that can also be used to make albumin hydrogels suitable for cells growth and proliferation [37,38,39]. Similar to other protein-derived polymers, albumin-based hydrogels can be printed and photo-crosslinked to provide a 3-dimensional scaffold suitable for cell proliferation and maintenance. Methacryloyl or methacrylate can be used to preserve the natural structure of albumin while performing photo-crosslinking [40,41]. The amount of methacrylate affected the pore size and the swelling behavior of albumin hydrogel [41]. Moreover, there was an inverse correlation between the amount of methacrylate and the degree of degradability of hydrogel [41]. Natural photo-initiators such as riboflavin and L-arginine were also used for albumin hydrogel photo-crosslinking [42].

In addition to cross-linked albumin films, nanoparticles and microspheres can also be prepared using coacervation methods, and cross-linking agents such as glutaraldehyde covalently stabilize microspheres [43,44] (Figure 3). Heat may be used for microsphere dehydration which results in the denaturation of the protein [45], whereas pH-coacervation could also be used to make albumin nanoparticles [46,47]. Chemical modification such as introducing methacrylic groups can be applied to albumin microspheres while maintaining the natural structure of albumin [45,48]. The supercritical antisolvent process is a micronization technique that does not require high temperature; therefore, the natural structure of the protein can be preserved [49]. This is the method of choice for encapsulating drugs and compounds with poor solubility [49]. Enzymatic polymerization of albumin can also be utilized using transglutaminase bacterial enzyme [5]. This enzyme cleaves the disulfide bonds and gelation of albumin happens in reducing environments [50]. Using this enzyme, albumin scaffolds with mechanical properties similar to collagen were prepared [5].

### 3.2. Mechanical Properties of Albumin Gels

**Figure 4:**
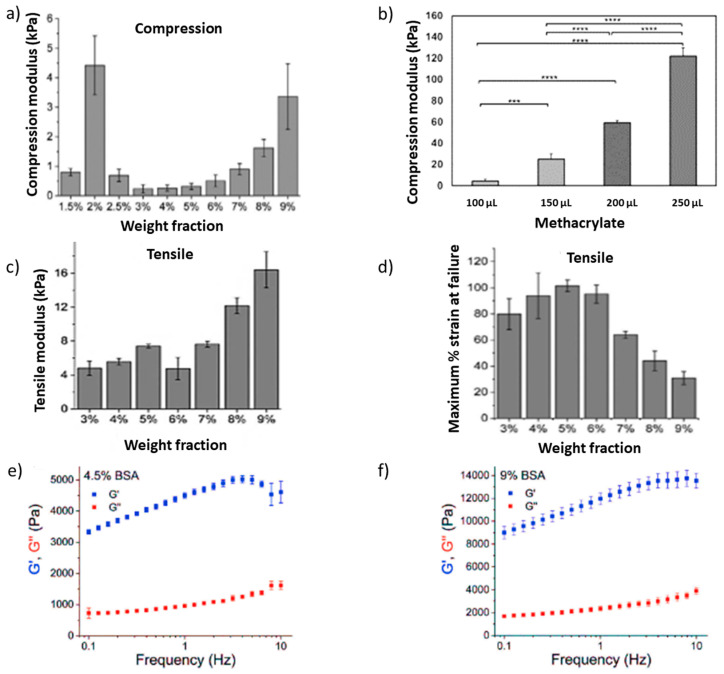
Mechanical properties of albumin hydrogel. (**a**) The effect of albumin concentrations on the compression modulus of hydrogels [30]. (**b**) The compression modulus is affected by the addition of different amounts of methacrylate to 5 mL of 20% albumin solution (*w*/*v*) and by the level of cross-linking [41] **** *p* < 0.0001, *** *p* < 0.001. (**c**,**d**) show the tensile strength of hydrogels containing different amounts of albumin [30]. Frequency-dependent rheological properties of hydrogels containing 4.5% (**e**) versus 9% (**f**) albumin [30]. G’: storage modulus G’’: loss modulus.

Several assessments can be used to measure the mechanical properties of hydrogels (Figure 4). Compression test measures the amount of compressive pressure that a hydrogel can tolerate before the disintegration of its structure [51,52]. Tensile testing or extensiometry measures the hydrogel viscoelastic characteristics [51,52]. In rheological analysis, the shear storage modulus (elastic response) and loss modulus (viscous response) are measured (Figure 4e,f). This analysis determines how a hydrogel behaves in response to stress over a period of time [53]. The mechanical strength of albumin hydrogels is affected by various parameters such as porosity, the degree of cross-linking, and the stiffness of the hydrogels. Increasing albumin concentration enhanced the mechanical strength of hydrogels since a denser network with less porosity is formed [33]. However, too-dense hydrogel structures resulted in a reduction in mechanical properties [33]. Similarly, the concentration of albumin solutions affects the elasticity of hydrogels [33]. Besides the albumin protein concentration, temperature and pH both can affect the mechanical properties of the formed hydrogels. Comparing the Young’s modulus of albumin hydrogels (20 wt%) formed at acidic conditions (pH 3.5, 37 °C) with thermally induced hydrogels (80 °C, pH 7.4) indicated that thermally induced hydrogels were stronger than pH-induced non-covalent hydrogels [31]. When the albumin hydrogel was formed at acidic pH 2 followed by heating at 75 °C, the elasticity of the gel was affected by concentrations of albumin. Albumin at a concentration ≤ 6 wt% produced a very elastic hydrogel, while increasing the albumin concentration resulted in the formation of inelastic and stiff hydrogels [30] (Figure 4c,d). Crosslinkers such as methacrylate can increase the mechanical strength of albumin hydrogels, and dual cross-linking using chemical reagents and heat resulted in a hydrogel with stronger mechanical properties compared with only chemically or physically cross-linked hydrogels [41,54] (Figure 4b).

## 4. Electrospun Albumin Fibers

Electrospinning is a versatile technology for the formation of fibers with a diameter in the range of nanometers from natural or synthetic polymers. Since fibers are electrospun in the absence of heat, electrospinning is an ideal method to encapsulate bioactive molecules while preserving the functionality and stability of proteins or drugs. However, the application of harsh solvents during the spinning process may be a limitation for electrospinning as denaturation may happen in some proteins and active molecules. During electrospinning and electrospraying, a high-voltage electric field is imposed on solutions of polymers or any other active ingredients and converts them into fine jets. These charged liquids either form fibers (electrospinning) or particles (electrospraying). In electrospinning, the spinneret through which the solution will be extruded is connected to the high voltage power supply, either negative or positive charge, and the collector is usually grounded or has an opposite charge to the spinneret [55]. As the spinnable solution is extruded from the spinneret and acquires charges, the solution surface tension and the potential differences between the solution and the collector determine whether the solution forms droplets or the Taylor cone [56]. If the Taylor cone forms, a jet of solution elongates towards the collector. During this elongation process, the solvent evaporates and as the jet reaches the grounded collector as fibers. Apart from the applied voltage, solution-related parameters such as viscosity, conductivity, surface tension, concentration, and volatility have important roles in determining fiber formation [56].

**Figure 5:**
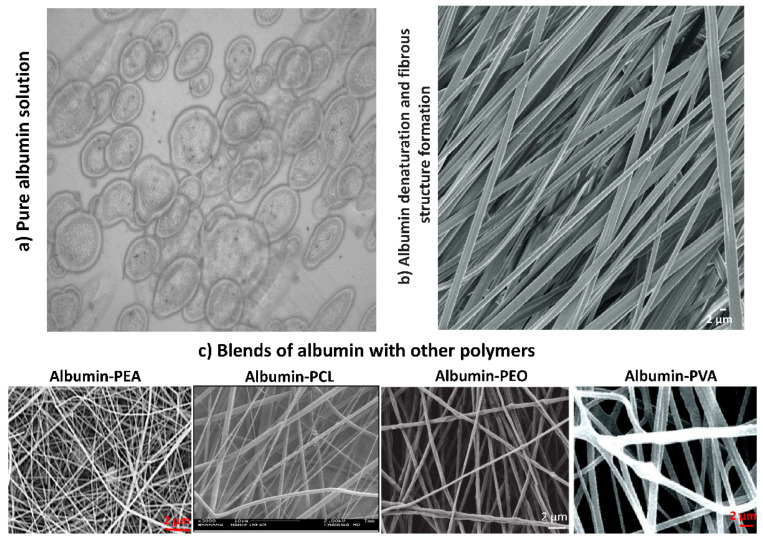
Electrospinning of albumin. (**a**) Inverted microscope image showing electrosprayed droplets from electrospinning of a pure albumin solution (unpublished image from the authors’ lab). (**b**) Denaturing solvents and reducing agents transform the albumin structure into electrospinnable fibrous structures [57]. (**c**) Electrospinning albumin into nanofibers can be achieved with polymers such as PEA (the SEM image contains unpublished data from the authors’ lab), Albumin-PCL [69], Albumin-PEO [61], and Albumin-PVA [67]. PEA: poly(ester amide), PCL: polycaprolactone, PEO: Polyethylene oxide, PVA: polyvinyl alcohol. Images from the cited references are used by permission.

Albumin is a globular protein, and its solution is conductive. Besides the molecular structure, for some types of albumins such as egg albumin, the molecular weight is low, so molecular entanglement does not easily happen during the electrospinning of pure albumin solutions. Because of the high viscosity, an increase in the albumin solution concentration results in non-spinnable solutions (Figure 5a). To convert albumin into an electrospinnable form, different strategies can be applied. One strategy is to transform the albumin globular structure into fibrous arrangements by means of denaturation which results in losing the globular structure into a fibrillary form (Figure 5b). Solvents such as trifluoroethanol unfold albumin native structure by preventing the hydrophobic interactions within the proteins [57] and further addition of β-mercaptoethanol (βME) reduces the disulfide bonds and results in an electrospinnable structure of albumin [57,58,59]. Albumin fibers electrospun using this method were shown to have similar mechanical properties to elastin and supported the adhesion of fibroblasts, muscles, cardiac, and endothelial cells in vitro [58,59]. When implanted subcutaneously, albumin fibers were degraded around 50 percent within three weeks with reported weak inflammation and fibrosis at the site of implantation [59]. Cationization of albumin decreases protease degradation and improves cell adhesion to fibers [60]. Moreover, positively charged amine groups on fibers enhanced cell adhesions attributed to positively charged fibers absorbing proteins and soluble factors supporting osteogenic differentiation of mesenchymal stem cells [60].

Blending of albumin with other polymers is another strategy to make albumin-modified fibers (Figure 5c). For instance, blending of albumin with poly(ester amide)s (PEA) results in the formation of electrospun nanofibers, while the electrospinning of albumin solution alone results in the electrospraying of albumin droplets (unpublished data) (Figure 5c). Polyethylene oxide (PEO) was used in combination with egg albumen as a fiber-forming agent (Figure 5c). It was shown that at the pH near the protein isoelectric point, bead formation happened in fibers, while neutral pH resulted in beads-free fibers [61]. Fluorescein isothiocyanate (FITC)-tagged albumin/PEO electrospun fibers were found to be pH sensitive and had the capability to be used as pH sensors [62]. Fluorescence intensity varied with alkaline pH due to changes in albumin secondary structure and small FRET (fluorescence resonance energy transfer) between fibers [62,63]. Albumin hydrogels were also shown to be pH-responsive [64] where a reversible pH-responsive behavior of BSA molecules adsorbed at the gold-saline interface behaves as a pH-sensitive sponge for water molecules to adsorb and desorb depending on the surrounding pH, at between 4 and 8 [64]. Human albumin and 25% PEO were used to prepare water-soluble fibers that after long-term aging at 37 °C, they were converted into insoluble albumin mats. In this combination, albumin preserved its natural properties such as being non-adherent to cells and were degraded in within 6 days in vivo [65]. Albumin in its native form is non-adhesive, however, irradiation and adsorption of cationic solutions such as polyethyleneimine (PEI) convert albumin from non-adherent to adherent forms [66]. In addition to PEA and PEO, polyvinyl alcohol (PVA) [67] and polycaprolactone (PCL) [68,69] can be blended for fabricating albumin nanofibers (Figure 5c). Protein release from albumin-PCL fibers occurred during the first 20 min of incubation in the PBS buffer and no further protein release was detected afterward. Albumin was detectable on the surface of fibers and was recognizable by cell receptors [68]. Compared with PCL alone, fibers containing albumin were more suitable for the proliferation of fibroblasts [70].

To confirm the presence and the distribution of components in a single fiber, different methods such as fluorescence microscopy, Energy-dispersive X-ray spectroscopy (EDX), and Raman spectroscopy [71] can be used. For fluorescence microscopy fiber analysis, albumin should be labeled with a fluorescent dye such as rhodamine B or fluorescein isothiocyanate before electrospinning [62,71]. This method is also suitable for release studies since the amount of protein released from fibers can easily and precisely be determined by measuring the fluorescent signal in the buffer using a fluorometer [72,73]. EDX analysis that is performed in conjunction with electron microscopy can map the fibers and give information about the surface elemental composition. The characteristics of the generated X-rays that are element-specific reveal the fiber composition [74]. Raman spectroscopy provides information about the chemical structures of fibers based on the properties of scattered laser lights after interaction with chemical bonds. This shows the components ratio of blended fibers by measuring the ratio of characteristics peaks intensities that also helps to understand if phase separation happened during electrospinning [71].

## 5. Albumin in Biomolecule Delivery

Albumin-based scaffolds have potential for drug delivery as they are non-toxic. Albumin binds to medicinal compounds and affects their biodistribution and half-life inside the body [75]. Encapsulation of drugs with albumin protects them against environmental conditions such as oxidation and degradation and results in effective drug delivery with reduced side effects. Active molecules such as growth factors or cytokines can be loaded into albumin nanoparticles and therapeutic proteins can covalently bind to albumin. This improves the stability and pharmacokinetics of therapeutic proteins. Moreover, albumin scaffolds act as biosensing systems such as pH sensors for use in the controlled release of molecules such as growth factors. Albuferon which is interferon fused to albumin and Abraxane or albumin paclitaxel nanoparticles are examples of albumin-based drugs [76,77]. Albumin also serves cells as a source of energy and nutrients [78]. Various cell types such as fibroblasts, kidney cells, and tumor cells are reported to express albumin receptors [3]. Membrane receptors such as glycoproteins, neonatal FC receptors, cubilin, megalin, and calreticulin can act as albumin receptors [3]. Albumin also has secreted receptors such as secreted protein acidic and rich in cysteine [3,79]. These receptors are involved in the uptake and catabolism of albumin in both normal and cancerous cells [3]. Fibroblasts can actively metabolize albumin [80] which in turn stimulates cell division of fibroblasts [81]. The presence of leaky capillaries in tumors also helps albumin to accumulate in tumor environments. These are the rationale for using albumin as a carrier for anticancer drugs. Albumin reduces the rapid clearance of drugs and facilitates the internalization of drugs by cancerous cells [3]. However, the applications of albumin in drug delivery are not limited to cancers. Albumin-based drug delivery is also applicable in other conditions such as rheumatoid arthritis in which inflammatory environments of joints attract more albumin [80]. Albumin-based delivery of methotrexate was analyzed in a mouse model of arthritis. Albumin-Methotrexate was more effective in the suppression of arthritis compared with methotrexate alone. This effect was reported to be a result of higher accumulations of albumin-methotrexate in inflamed joints [80].

**Figure 6:**
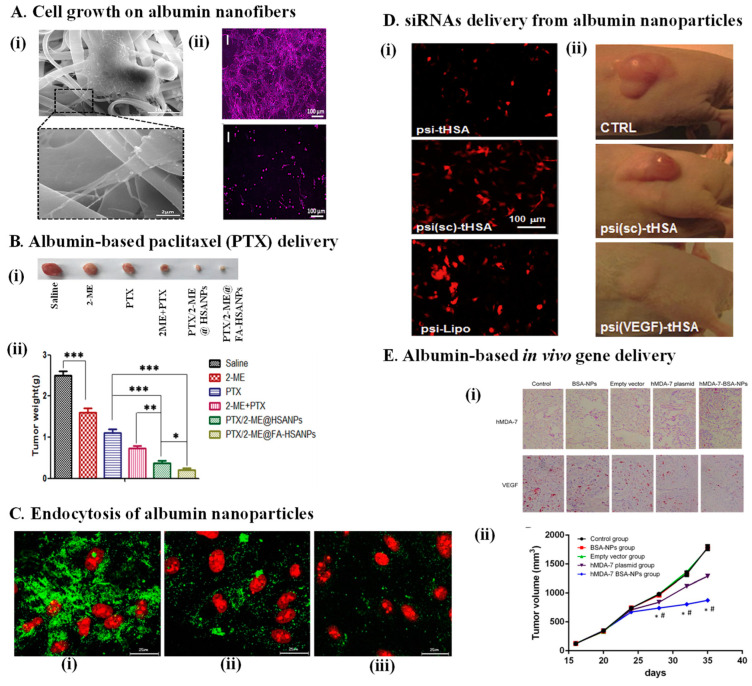
Applications of albumin scaffolds in regenerative medicine. (**A**). (**i**) SEM image of cardiomyocytes interaction with albumin fibers. (**ii**) Cultured cardiomyocytes on albumin fibers (top) and PCL fibers (bottom) (images taken from [58] with permission). (**B**). Improved paclitaxel (PTX) delivery using albumin nanoparticles. (**i**) the effect of treatments on tumor size after 13 days of daily intravenous injection of either saline, methoxyestradiol (2-ME), PTX, a combination of 2-ME and PTX, human serum albumin nanoparticles containing 2ME/PTX combination (PTX/2-ME @HSANPs), and folate-conjugated PTX/2-ME @HSANPs. (**ii**) Albumin nanoparticles and folate-targeted albumin nanoparticles significantly improved the treatment efficacy, * *p* < 0.05, significant; ** *p* < 0.01, very significant; *** *p* < 0.001, extremely significant (taken from [85] with permission). (**C**). Cells absorb albumin nanoparticles through caveloae- and clathrin- mediated endocytosis. (**i**) Confocal image of cellular uptake of albumin nanoparticles without chlorpromazine (clathrin inhibitor) or flipin (caveloae inhibitor); (**ii**) albumin nanoparticle cellular uptake in the presence of chlorpromazine (clathrin inhibitor) or flipin (caveloae inhibitor) (**iii**) (images taken from [92] with permission). (**D**). Effective siRNAs delivery using albumin nanoparticles. (**i**) Polymerized siRNAs (psi) were more efficiently delivered by albumin nanoparticles (psi-tHSA) than by transfection reagents (psi-Lipo); (**ii**) treatment with psi-tHSA resulted in the targeted silencing of VEGF expression and a significant reduction in the tumor size. Psi-sc-tHSA: scrambled siRNA-tHSA (taken from [94] with permission). (**E**). In vivo gene delivery using albumin nanoparticles. In comparison with different control groups, albumin nanoparticles containing the hMDA-7 gene (hMDA-7-BSA-NPs) significantly reduced the expression of vascular endothelial growth factor (VEGF) (**i**) in tumor tissues and decreased the size (**ii**) of pancreatic xenograft tumors. NPs: nanoparticles, BSA: bovine serum albumin # *p* <0.05 compared to the hMDA-7 plasmid group (images are taken from [96]) under the terms of the Creative Commons Attribution License.

Different types of albumin-based scaffolds are used for drug delivery. Albumin-based fibers help prolonged drug release. Moreover, the degree of biodegradation of fibers can be manipulated based on the amount of protein [82]. For example, tetracycline hydrochloride was loaded on albumin/PVA nanofibers or albumin/PCL nanofibers which were used for controlled release of nerve growth factor over a period of 28 days [67,69]. This effect was not achieved with pure PCL nanofibers [69]. Besides nanofibers, albumin microspheres and nanoparticles also have the potential as drug delivery systems. For instance, the controlled release of small molecules was achieved using PEG-conjugated albumin hydrogels [35]. Methacrylate derivatized albumin was copolymerized with methacrylic acid using reverse-phase suspension copolymerization to create pH-sensitive albumin hydrogel microspheres suitable for drug delivery and pH-sensitive release of drugs in the oral cavity [83]. Albumin nanoparticles loaded with noscapine were designed as a potential delivery system in breast cancer [46]. Terbutaline sulfate which is a cough medicine was delivered using albumin microspheres to achieve more lung-specific delivery of medicine [44]. Albumin nanoparticles containing ganciclovir or meloxicam were produced to increase the half-life and bioavailability of drugs [43,47]. Albumin nanoparticles also have applications in protein delivery such as albumin nanoparticles containing antibacterial enzymes [84]. Ligands can be added to albumin nanoparticles to achieve more targeted drug delivery [85,86]. For example, since many cancerous cells express folic acid receptors, folic acid-conjugated albumin can be used for making drug-containing albumin nanoparticles to increase cellular uptakes of nanoparticles by cancerous cells [49,85] (Figure 6B). Some albumin-based drugs such as Abraxane are approved by the US Food and Drug Administration [87]. Table 1 provides a summary of selected albumin-based drug delivery systems. A search of clinical trials (https://clinicaltrials.gov, accessed on 12 September 2022) on albumin-based drug delivery revealed several studies investigating Abraxane’s efficacy and safety. We found 66 clinical studies on Abraxane with available results, of which 15% are in phase I, 71% were in phase II, and 14% have completed phase III clinical trials. Studies have assessed the efficacy and safety of Abraxane alone or in combination with other drugs for the treatment of various types of cancers, including breast, melanoma, pancreatic, ovarian, cervical, bladder, colorectal, and lung cancers. Albuferon’s effectiveness in treating hepatitis was also evaluated in phase 3 clinical studies registered on the European clinical trial registry (https://www.clinicaltrialsregister.eu/, accessed on 6 October 2022).

## 6. Albumin in Cell Delivery and Tissue Engineering

Targeted delivery of cells to a specific organ or tissue and preserving the cellular functions are requirements for successful cell therapies. Local or systemic injections of cells suffer from some limitations such as unwanted delivery of cells to nonspecific tissues, lack of engraftments, and cells survival. To overcome some of these limitations, synthetic or natural scaffolds are designed as cell carriers. Natural scaffolds such as albumin scaffolds resemble natural extracellular matrices and can provide biological cues essential for cellular maintenance and functions. Therefore, scaffold-based cell delivery has emerged as an alternative strategy to improve the delivery and maintenance of donor cells. Besides the in vivo delivery of cells, scaffolds derived from natural proteins have broad applications in the reconstruction and engineering of tissues. These scaffolds provide three-dimensional milieus to grow cells in environments similar to inside the body. Not surprisingly, albumin as one of the main natural proteins with the capability to attach to various cell types has been successfully used in cell delivery and tissue engineering. For example, a glutaraldehyde-crosslinked albumin scaffold was used to successfully deliver adipose-derived stem cells and olfactory ensheathing cells to the site of spinal cord injury in rats [88]. Immunohistological analysis showed that albumin scaffold by itself played a significant role in reducing the scar size and the delivery of cells leading to tissue repair [88]. A foam-type albumin scaffold fabricated from a mixture of albumin and starch using a freeze-drying technique had a porous and stable structure without toxicity for seeded cells [89]. Electrospun bovine albumin scaffolds that were functionalized with growth factors and iron-coating porphyrin to impart electrical conductivity were used for neural tissue engineering [90]. This scaffold could support the proliferation and differentiation of neural stem cells. While the electrical conductivity of the scaffold induced the maturation of neural cells [90]. The potential of heat and pH-induced albumin hydrogel to protect cardiac myocytes has also been shown [30,58]. In addition to hydrogels, electrospun albumin fibers were shown to provide a suitable three-dimensional scaffold for the growth and contraction of cardiac cells where they had mechanical properties similar to cardiac tissue [58] (Figure 6A). Although bone engineering requires scaffolds with stronger mechanical properties [91], albumin was shown to support the proliferation of osteoblasts [37,39]. The culture of osteoblasts on albumin sponges prepared by cross-linking human blood-derived serum with glutaraldehyde resulted in mineralization and formation of bone-like structures [39]. To this end, human alveolar osteoblasts were seeded on glutaraldehyde cross-linked albumin hydrogels [37]. After the implantation of these cell-containing albumin scaffolds in animals, bone formation was observed 5 weeks post-surgery. Successful bone repair was also observed in animals that received glutaraldehyde cross-linked albumin scaffolds with or without adipose tissue-derived mesenchymal stem cells [38].

## 7. Albumin-Mediated Gene Delivery

Scaffold-based gene delivery could be a method of choice for non-viral gene delivery. This strategy allows more targeted delivery of genes as well as the controlled and sustained release of vectors at the target tissue. The utility of albumin nanoparticles to deliver the superoxide dismutase gene into human retinal pigment epithelial cells was studied [92]. The desolvation method was used to produce these vectors-containing albumin nanoparticles [92,93]. It was shown that these nanoparticles protected the gene from DNase I degradation. Albumin nanoparticles enter the cell via receptor-mediated endocytosis, and they can pass the nucleus membrane (Figure 6C). The efficiency of gene transfer was reported to be 80% and much higher than the lipofectamine facilitated gene delivery method [92]. Cationic albumin and double emulsion and solvent evaporation methods were also used to prepare pegylated albumin nanoparticles containing gene vectors [94]. The results from this study indicated that gene-containing albumin nanoparticles were able to pass the blood–brain barrier and translocate in the nucleus of brain tumor cells [94]. Nuclear localization signals were added to the vector-albumin complex to enhance the nuclear localization of the gene complex [95]. Desolvation and coacervation methods with or without glutaraldehyde cross-linking were used by several studies to prepare gene-containing albumin nanoparticles [96,97,98,99] (Figure 6E). The uptake and targeting of these nanoparticles can be enhanced by adding ligands at the surface of albumin nanoparticles [98,100]. To improve the complex formation between albumin and short anionic oligonucleotides such as siRNA, thiolated albumin was used [101,102]. This method resulted in the self-crosslinking of albumin nanoparticles [101] (Figure 6D). Another approach to use albumin for gene delivery was introduced by a study that incorporated an albumin binding sequence to an adenovirus vector. Upon entry to the blood, the vector bound with serum albumin and its delivery to liver tissue was significantly increased [103]. All of these studies showed successful albumin-mediated gene transfer either in vitro or in vivo.

## 8. Egg White as a Rich Source of Albumin

**Figure 7:**
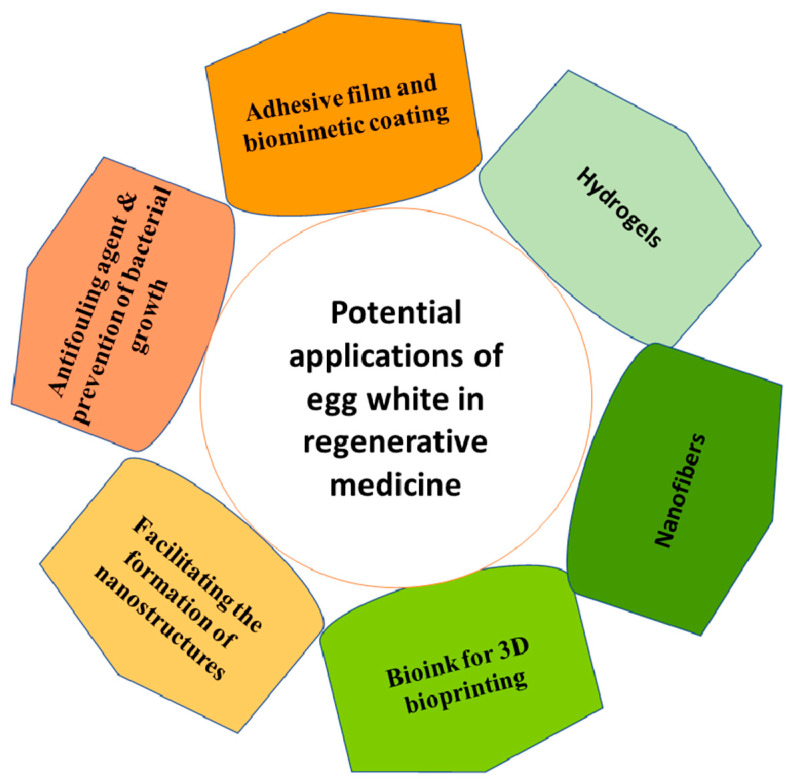
Different applications of egg white in regenerative medicine. As a rich source of albumin, egg white can be used to fabricate scaffolds such as hydrogels and nanofibers. It can be used as a bioink and coating agent with antibacterial properties. Moreover, egg white has antifouling properties similar to albumin.

Chicken egg white, composed of at least 158 different proteins, is an interesting biomaterial. Ovalbumin, lysozyme, ovomucin, ovomucoid, ovotransferrin, avidin, ovosecretoglobin with a function similar to α-2-macroglobin, ovostatin, chondrogenesis-associated lipocalin, apolipoprotein-D, ovoglycoprotein, and defensin were among the list of detected proteins [104,105]. Although the functions of most egg white-derived proteins are not fully understood, some such as albumin have emerging biomedical applications. Therefore, egg white as an accessible, low-cost, and easy to process albumin source has attracted considerable attention in various industries from edible packaging films to bioscaffolds as recently published [106,107]. The presence of free thiol groups in ovalbumin has an important role in film formation from egg white [106]. When mixed with plasticizers such as glycerin, egg white resembles a bioplastic [108]. Egg white is also a good matrix for the production of biomimetic films such as biomelanin [106]. Since egg white has a high dielectric constant, it has diverse applications in organic electronics [109]. In biomedical research, egg white-derived hydrogels have various applications in cell and drug deliveries (Figure 7). A combination of chicken egg white and sodium alginate was used as bioink for bioprinting of cells. In this construct, sodium alginate improved the rheological properties of bioink to be more printable and egg white enhanced the biocompatibility of the bioink [110]. These types of egg white alginate hydrogels were successfully used as a three-dimensional matrix to culture organoids [40].

The gelation of egg white can be achieved at alkaline conditions (pH~13) and room temperature, a condition that resulted in the self-assembly of highly stretchable gels suitable for 3D bioprinting [111]. Alternatively, a stable gel with a homogenous structure can be formed by heating egg white at alkaline pH ~9, while at acidic pH ~5, the gel is loose and nonhomogeneous [112]. However, ovomucin alone which is the gelling agent of egg white could form a gel at room temperature and neutral pH [113]. Since ovomucin is a weak polyelectrolyte, electrostatic interactions are important to have a stable ovomucin gel [113]. Combining egg white with surfactant resulted in the formation of protein condensate which was mainly composed of ovalbumin and a strong gel was formed after heating this protein condensate at 70 °C for 20 min. The formed gel was elastic with a 150-fold higher fraction strength than boiled egg white resulted from covalent disulfide bonds between the thiol groups of the heat-denatured albumin protein [114]. Another study showed that the modification of lysyl groups of egg white protein and incorporation of carboxylic groups increased the swelling ratio of egg white-derived hydrogel [115]. A combination of egg white and PVA was used to fabricate hydrogels suitable for wound dressing with a good water absorption capacity [116]. Clay nanoparticles were incorporated into the hydrogel to improve its thermal stability [116]. Furthermore, honey was loaded into clay nanoparticles and nanocomposite hydrogels were formed from egg white and PVA with the capability to control the moisture of the wound thereby enhancing the healing process [117]. PVA and egg white dissolved in formic acid (85%) was also used to electrospun non-woven nanofibers [118]. Photopolymerized egg white/eggshell-based hydrogels were made with a porous structure suitable for osteogenic differentiation of human stem cells [119]. The ovomucin-based hydrogel was shown to be biocompatible, and its sub-cutaneous implantation did not activate the host immune response [120]. Similar to albumin, egg white can be used as an antifouling agent; hence, coating a layer of egg white on the surface of implants prevented the adhesion of bacteria such as *Staphylococcus aureus* on biomaterial surfaces and reduced the biofilm formation [121]. Not only albumin but also the ovomucin protein in egg white has been shown to have antifouling properties [122]. Egg white facilitates the formation of nanostructures such as zinc oxide nanorods [123], enabled encapsulation of drug-containing nanoparticles with egg white protected the nanoparticles from degradations [106] and egg white nanoparticles showed potential as drug delivery vehicles to target cancer cells [124]. Egg white could form hydrogen bonds with hyaluronic acid that resulted in the formation of new thin adhesive films [125]. These films can have applications in drug delivery and biomimetic coatings [125].

## 9. Conclusions and Future Perspectives

The physiological role of serum albumin as a major modulator of plasma oncotic pressure and transporter of drugs and ligands has long been known. Over the past few decades, this plasma protein has also emerged as a candidate for biomedical applications taking advantage of its biodegradable and biocompatible properties and the fact that it does not elicit an immune reaction. As discussed in this article, recent studies have shown that albumin is very promising as a suitable material in the biomedical field. More specifically, overexpression of albumin receptors in tumors has made albumin a promising carrier for cancer drug delivery; however, to increase the chance of targeted drug delivery, the surface of the albumin scaffold may need to be functionalized using tissue-specific ligands or receptors. Among different organs, the delivery of therapeutic agents to the brain and eyes is faced with several limitations. Albumin is already being used as a tear supplement to treat dry eye; however, only limited studies have shown the capacity of albumin to deliver drugs or genes inside the ocular cavity or across the blood–brain barriers. More studies may lead to a new solution to successful brain or intraocular drug/gene deliveries.

Expanding their utilities in the biomedical field, gels and fibrous mats may be fabricated from albumin, although the fabrication of fibrous mats from pure albumin is somewhat challenging. Future studies that fabricate albumin hydrogel nanofibers may provide novel insights into their applications as a skin substitute since the structures resemble dermal and epidermal layers of skin. Due to its tunable properties, it is possible to fabricate albumin materials with viscoelasticity approaching human skin, and the conductivity of albumin may facilitate skin neurogenesis. The abundant supply of albumin from chicken egg white is affordable instead of pure albumin without requirements for protein isolation. Taken together, established and emerging data strongly favor the future of albumin biomaterials.

## Figures and Tables

**Table 1:** Application of albumin in drug delivery.

Delivery System	Loaded Drug	Type of Study	Key Findings	Refs
Albumin nanoparticles	Interferon alpha (Albuferon^®^)	Clinical trial	Significant antiviral activity with mild adverse effects similar to interferon alphas alone	[76]
Albumin nanoparticles	Paclitaxel (Abraxane^®^)	Clinical trial (FDA approved)	Reduced the toxicity of Paclitaxel	[87]
Albumin- methotrexate conjugate	Methotrexate	Preclinical	Enhanced delivery of drug to the arthritic joints More effective suppression of arthritis	[80]
Albumin/PVA nanofibers	Tetracycline hydrochloride	Preclinical	Controlled release of drug	[67]
Albumin/PCL nanofibers	Nerve growth factor	Preclinical	Prolong controlled release of drug	[69]
PEG-conjugated albumin hydrogels	Warfarin and Naproxen	Preclinical	Controlled release of small molecules	[35]
pH-sensitive albumin hydrogel microspheres	β-Propranolol	Preclinical	Efficient release of drugs in the pH similar to oral cavity	[83]
Albumin nanoparticles loaded with noscapine	Noscapine	Preclinical	Enhanced effectiveness on breast cancer cells	[46]
Albumin microspheres	Terbutaline sulfate	Preclinical	lung-specific delivery of medicine	[44]
Albumin nanoparticles	Meloxicam	Preclinical	Targeted delivery to inflamed tissues. Sustained release and increased half-life and bioavailability of drugs	[47]
Albumin nanoparticles	Ganciclovir	Preclinical	Sustained release of drugs	[43]
Folic acid conjugated albumin nanoparticles	10-Hydroxycamptothecin and paclitaxel/2-methoxyestradiol	Preclinical	Prolonged release, increased uptake by cancerous cells and targeted inhibition of tumor growth	[49,85]

## Data Availability

Not applicable.
